# Diagnostic accuracy of quantitative neuromuscular ultrasound for the diagnosis of intensive care unit-acquired weakness: a cross-sectional observational study

**DOI:** 10.1186/s13613-017-0263-8

**Published:** 2017-04-05

**Authors:** Esther Witteveen, Juultje Sommers, Luuk Wieske, Jonne Doorduin, Nens van Alfen, Marcus J. Schultz, Ivo N. van Schaik, Janneke Horn, Camiel Verhamme

**Affiliations:** 1 Department of Intensive Care Medicine, Academic Medical Center, Meibergdreef 9, 1105 AZ Amsterdam, The Netherlands; 2 Laboratory of Experimental Anesthesiology and Intensive Care (L·E·I·C·A), Academic Medical Center, Amsterdam, The Netherlands; 3 Department of Neurology, Academic Medical Center, Amsterdam, The Netherlands; 4 Department of Rehabilitative Medicine, Academic Medical Center, Amsterdam, The Netherlands; 5 Department of Neurology and Clinical Neurophysiology, Donders Center for Neuroscience, Radboud University Medical Center, Nijmegen, The Netherlands; 6 Department of Intensive Care Medicine, Radboud University Medical Center, Nijmegen, The Netherlands

**Keywords:** ICU-AW, Critical illness myopathy, Critical illness polyneuropathy, Critical illness neuromyopathy, Neuromuscular ultrasound, Diagnosis

## Abstract

**Background:**

Neuromuscular ultrasound is a noninvasive investigation, which can be easily performed at the bedside on the ICU. A reduction in muscle thickness and increase in echo intensity over time have been described in ICU patients, but the relation to ICU-acquired weakness (ICU-AW) is unknown. We hypothesized that quantitative assessment of muscle and nerve parameters with ultrasound can differentiate between patients with and without ICU-AW. The aim of this cross-sectional study was to investigate the diagnostic accuracy of neuromuscular ultrasound for diagnosing ICU-AW.

**Methods:**

Newly admitted ICU patients, mechanically ventilated for at least 48 h, were included. As soon as patients were awake and attentive, an ultrasound was made of four muscles and two nerves (index test) and ICU-AW was evaluated using muscle strength testing (reference standard; ICU-AW defined as mean Medical Research Council score <4). Diagnostic accuracy of muscle thickness, echo intensity and homogeneity (echo intensity standard deviation) as well as nerve cross-sectional area, thickness and vascularization were evaluated with the area under the curve of the receiver operating characteristic curve (ROC–AUC). We also evaluated diagnostic accuracy of *z*-scores of muscle thickness, echo intensity and echo intensity standard deviation.

**Results:**

Seventy-one patients were evaluated of whom 41 had ICU-AW. Ultrasound was done at a median of 7 days after admission in patients without ICU-AW and 9 days in patients with ICU-AW. Diagnostic accuracy of all muscle and nerve parameters was low. ROC–AUC ranged from 51.3 to 68.0% for muscle parameters and from 51.0 to 66.7% for nerve parameters.

**Conclusion:**

Neuromuscular ultrasound does not discriminate between patients with and without ICU-AW at the time the patient awakens and is therefore not able to reliably diagnose ICU-AW in ICU patients relatively early in the disease course.

**Electronic supplementary material:**

The online version of this article (doi:10.1186/s13613-017-0263-8) contains supplementary material, which is available to authorized users.

## Background

Intensive care unit-acquired weakness (ICU-AW) is an important cause of morbidity in critically ill patients and develops in approximately 50% of patients in the ICU [1, 2]. Patients with ICU-AW suffer from severe weakness affecting all extremities and often fail to wean from the ventilator [3]. ICU-AW is caused by muscle dysfunction (critical illness myopathy; CIM), nerve dysfunction (critical illness polyneuropathy; CIP) or a mixed dysfunction (critical illness neuromyopathy (CINM) [3].

ICU-AW is diagnosed by assessment of manual muscle strength, using the Medical Research Council (MRC) score [3, 4]. A major limitation of muscle strength testing is that patients need to be awake and cooperative for reliable assessment [5]. Since consciousness and cooperativeness are often impaired in ICU patients, especially in the first days after ICU admission, a diagnosis of ICU-AW is often delayed [6]. To diagnose ICU-AW at an early stage, other diagnostic methods are needed.

Neuromuscular ultrasound (NMUS) is an upcoming technique to diagnose muscle disorders [7] and peripheral neuropathies [8]. NMUS can detect muscle atrophy and changes in muscle architecture. Muscle echo intensity may increase due to an increase in fat and fibrous tissue [7]. It can be quantified with computer software by calculating the average grayscale level of the muscle, which is more accurate and objective than visual evaluation [9]. Nerve cross-sectional area (CSA) and echo intensity can also be quantified, as well as increased intraneural vascularization [10].

A limited number of muscle ultrasound studies have been performed in ICU patients, which were recently summarized in two systematic reviews [11, 12]. A reduction in muscle thickness [13–20] or CSA [17, 21] and increase in echo intensity [16, 17, 22] over time are reported. As these studies did not discriminate between patients with and patients without ICU-AW, it is unknown whether these changes are specific for ICU-AW and can be used to diagnose ICU-AW, or that these changes are found in all ICU patients. Nerve ultrasound parameters have never been assessed in ICU patients [11].

The aim of this cross-sectional study was to investigate the diagnostic accuracy of quantitative NMUS for diagnosing ICU-AW. We hypothesized that quantitative NMUS can discriminate between patients with and without ICU-AW at the time the patient awakens.

## Methods

### Design and ethical approval

This cross-sectional observational study was performed in the mixed medical–surgical ICU of the Academic Medical Center, Amsterdam, The Netherlands, and was designed in accordance with the STARD criteria [23].

The Institutional Review Board of the Academic Medical Center Amsterdam approved the study (NL41156.018.12.; 2012_264 #B2013585a), and the study was registered in the Netherlands Trial Register (NTR4148). Informed consent was obtained from all patients prior to inclusion.

### Inclusion and exclusion criteria

Consecutive, newly admitted ICU patients, who were mechanically ventilated for ≥48 h, were eligible for inclusion. Patients with an admission diagnosis of a neuromuscular disorder, stroke, cardiac arrest, traumatic brain injury, spinal injury, or intracerebral infection or space-occupying lesion were excluded. In addition, we excluded patients with a poor pre-hospital functional status (modified Rankin >3) [24], preceding spinal injury and patients in whom no arms or no legs were available for muscle strength testing or ultrasound.

### Medical Research Council score (the reference standard)

Muscle strength was assessed as soon as patients were awake [Richmond Agitation Sedation Scale (RASS) between −1 and 1] and cooperative (able to follow 5 verbal commands with facial muscles, as scored by the Score of 5 Questions [24]). Assessment was done by trained and experienced physiotherapists who were blinded for ultrasound results. The MRC score was used for assessment of strength in the following six muscle groups bilaterally: wrist dorsiflexors, elbow flexors, shoulder abductors, hip flexors, knee extensors and ankle dorsiflexors. ICU-AW was defined as a mean MRC score <4, in accordance with the international consensus statement [3].

### Neuromuscular ultrasound measurements (the index test)

On the same day of the muscle strength assessment, NMUS testing was done by trained assessors (EW or CV) who were blinded for the muscle strength results, using an Esaote MyLabTwice ultrasound machine (Esaote, Genova, Italy). The biceps brachii (BB) muscle, tibialis anterior (TA) muscle and median nerve were measured on the left side of the body, and the rectus femoris (RF) muscle, flexor carpi radialis muscle (FCR) and peroneal nerve on the right side. If ultrasound was not possible on the preferred side, for example due to arterial lines or dressings, the opposite side was studied.

For muscle assessment, a 4–13 MHz linear array transducer was used with constant image acquisition settings, including constant focus. Three independent transverse images were taken per muscle based on predefined anatomical landmarks (Additional file 1: Figure E1; Additional file 1: Table E1). For assessment of muscle thickness, depth settings were adapted, if needed.

For quantitative echo intensity analysis, ultrasound images were analyzed with an in-house developed software routine for MATLAB (R2014b, Mathworks, Natick, MA, USA). Briefly, regions of interest (ROIs) for echo intensity measurements were drawn within the muscle following the contours of the muscle just below the fascia (Additional file 1: Figure E2). The lateral borders of the ROI were removed to exclude artifacts on the border of each image. The average echo intensity of the three images was used for analyses. The standard deviation (SD) of the average gray scale levels, as a measure of homogeneity, was analyzed as described before [16]. A more homogenous muscle may be caused by loss of muscle architecture due to muscle breakdown, inflammation or fluid retention [16].

Muscle thickness and echo intensity can be age, gender, dominance, length and weight dependent [25]. Therefore, raw ultrasound values were converted to *z*-scores using normal values which were acquired from published healthy populations [25, 26] and a new healthy population (see Additional file 1: Table E2 for the regression model formulas and Additional file 1: Table E3 for the characteristics of the new healthy control population). The *z*-scores represent the distance between the raw ultrasound value and the healthy population mean in units of the standard deviation. A *z*-score is negative when the raw score is below the population mean and positive when above.

For nerve assessment, a 6–18 MHz linear array transducer was used with constant image acquisition settings. Focus was adjusted according to nerve depth. Intraneural vascularization was investigated with power Doppler (low pulse repetition frequency 500 Hz, frequency 11.1 MHz, Doppler gain adjusted until random noise was encountered and then lowered until the noise disappeared, low persistence [27]). The median nerve was assessed at the wrist and 7 cm proximally and the peroneal nerve at the fibular head and at the popliteal fold, in a transversal and longitudinal plane. On transverse images, the cross-sectional area was measured within the hyperechogenic rim. Nerve diameter thickness was assessed on longitudinal images.

### Clinical data collection

We collected the following clinical characteristics: age, sex, body weight and length at ICU admission, hand dominance, admission type, admission diagnosis, Acute Physiology and Chronic Health Evaluation IV (APACHE IV) score, maximal total Sequential Organ Failure Assessment (SOFA) score and the presence of sepsis (according to the Bone criteria [28]) before inclusion. In addition, we collected data on pre-existing polyneuropathy or myopathy, risk factors for polyneuropathy before ICU admission (diabetes mellitus, alcohol abuse, chemotherapy, kidney failure), days with mechanical ventilation, ICU length of stay and ICU mortality.

### Sample size estimation

Sample size calculation was based on results of one study, in which echo intensity of the TA muscle increased by a factor 1.2 between 0 and 14 days after ICU admission [16]. We used the standardized echo intensity of the TA for males (33.9 SD 9.3) [25] and multiplied this value by factor 1.2, giving 40.8, again assuming a SD of 9.3. Thirty patients per group were required to detect a difference of 6.9 (40.8–33.9) with 80% power and a two-sided alpha level of 0.05. Incidence of ICU-AW in patients mechanically ventilated for >48 h in our institution is around 50% [29, 30]. To account for technically imperfect data, we aimed to include at least 70 patients.

### Statistical analysis

Normally distributed values are presented as mean with SD, non-normally distributed values as median with interquartile range (IQR), and proportions with percentages and total numbers. Differences between normally distributed continuous variables were assessed using Welch’s *t* test, and between non-normally distributed continuous variables using Wilcoxon rank-sum test. Pearson’s Chi-square or Fisher exact test was used to assess differences between proportions.

As a measure of variability, the median coefficient of variation (CV) of three analyses of echo intensity per muscle was assessed.

Discriminative power of NMUS was assessed using receiver operating characteristic (ROC) curves with calculated area under the curve (AUC) with 95% confidence interval (CI). Discriminative power of AUC values between 90 and 100% was defined as excellent, between 80 and 90% as good, between 70 and 80% as fair, between 60 and 70% as poor and <60% as failed.

Secondly, sensitivity and specificity and positive and negative predictive values (PPV, NPV) for muscle thickness and echo intensity were calculated based on a *z*-score cutoff of −2 for thickness parameters and +2 for echo intensity. We chose this cutoff point since 95% of the *z*-score values of healthy people lie between −2 and +2. Since weakness in ICU-AW is diffuse, we also investigated a composite outcome of amount of muscles with a thickness *z*-score of −2, or echo intensity of +2.

Analyses were done using R version 3.1.2 and R studio with the following packages: plyr, pROC, caret, tableOne, ggplot2.

## Results

**Fig. 1:**
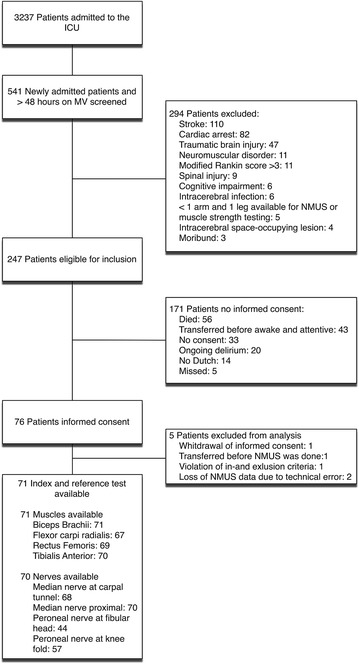
Flowchart of patient screening and inclusion. *ICU* intensive care unit, *MV* invasive mechanical ventilation, *NMUS* neuromuscular ultrasound

From September 1, 2013 to June 1, 2015, a total of 76 patients gave informed consent and 71 were available for analysis, of whom 41 had ICU-AW. See Fig. 1 for the flowchart of screening and inclusion. Patient characteristics are presented in Table 1.

**Table 1:** Patient characteristics

	No ICU-AW *N* = 30	ICU-AW *N* = 41	*P* value
Male (%)	22 (73)	25 (61)	0.41
Age (median years [IQR])	62 [49–69]	60 [51–70]	0.80
Body length (median cm [IQR])	175 [170–180]	172 [168–183]	0.46
Body weight (median kg [IQR])	75 [70–81]	75 [65–90]	0.66
History of myopathy (%)	0 (0)	1 (2)	1.00
History of polyneuropathy (%)	0 (0)	0 (0)	1.00
Any polyneuropathy risk factor in history (%)	14 (47)	22 (54)	0.73
Diabetes mellitus (%)	2 (7)	5 (12)	0.69
Alcohol abuse (%)	5 (17)	10 (24)	0.56
Kidney failure (%)	2 (7)	1 (2)	0.57
Chemotherapy (%)	7 (23)	9 (22)	1.00
Admission reason (%)			0.87
Medical (%)	12 (40)	18 (44)	
Emergency surgical (%)	9 (30)	10 (24)	
Elective surgical (%)	9 (30)	13 (32)	
Dominant hand right (%)	26 (90)	34 (92)	1.00
APACHE IV score (median [IQR])	58 [50–77]	71 [61–85]	<0.01
Maximum total SOFA score before ultrasound (median [IQR])	8 [8–12]	12 [10–14]	<0.01
Sepsis before ultrasound (%)	18 (60)	32 (78)	0.17
Mean MRC (median [IQR])	4.4 [4.0–4.7]	3.5 [2.8–3.7]	NA
Day of MRC (median [IQR])	7 [4–11]	9 [6–14]	0.02
Day of NMUS (median [IQR])	7 [5–10]	9 [6–14]	0.02
Length of MV (median days [IQR])	6 [3–8]	8 [5–17]	0.01
Length of stay on ICU (median days [IQR])	9 [6–13]	15 [9–23]	<0.01
Death in ICU (%)	0 (0)	2 (5)	0.51

*IQR* interquartile range, *APACHE* Acute Physiology and Chronic Health Evaluation, *SOFA* sequential organ failure assessment, *MRC* medical research council, *NMUS* neuromuscular ultrasound, *MV* mechanical ventilation, *ICU* intensive care unit

### Muscle ultrasound

Thickness and thickness *z*-scores were lower in BB and FCR muscles in ICU-AW patients compared to patients without ICU-AW (Table 2; Additional file 1: Figure E3). However, discrimination between patients with ICU-AW and patients without ICU-AW was poor for BB and FCR and failed for RF and TA thickness and all *z*-scores of thickness (Table 2).

**Table 2:** Univariate analysis of muscle thickness, echo intensity and echo intensity standard deviation (SD) and z-scores, and area under the receiver operating characteristic curve (ROC–AUC)

	No ICU-AW	ICU-AW	*P* value	ROC–AUC (95% CI)
Thickness				
BB thickness [mean cm (SD)]	2.6 (0.6)	2.2 (0.5)	0.002*	68.0 (55.4–80.7)
FCR thickness [mean cm (SD)]	1.1 (0.2)	1.0 (0.2)	0.035*	64.5 (50.4–78.7)
RF thickness [mean cm (SD)]	3.1 (0.9)	2.8 (0.8)	0.258	55.6 (41.8–69.4)
TA thickness [mean cm (SD)]	2.2 (0.4)	2.1 (0.4)	0.558	54.3 (40.3–68.3)
*Z*-score thickness				
BB *z*-score thickness [mean (SD)]	0.1 (0.6)	−0.3 (0.5)	0.011*	66.7 (53.7–79.6)
FCR *z*-score thickness [mean (SD)]	−0.7 (1.2)	−1.3 (0.9)	0.033*	65.5 (51.3–79.7)
RF *z*-score thickness [mean (SD)]	−0.5 (0.7)	−0.6 (0.8)	0.656	53.2 (39.4–67.1)
TA *z*-score thickness [mean (SD)]	−0.4 (0.7)	−0.4 (1.0)	0.818	51.3 (37.5–65.1)
Echo intensity				
BB absolute echo intensity [mean gray scale level (SD)]	76.2 (14.1)	79.1 (16.4)	0.434	57.6 (44.0–71.3)
FCR absolute echo intensity [mean gray scale level (SD)]	62.3 (12.6)	67.7 (13.6)	0.095	63.2 (49.6–76.9)
RF absolute echo intensity [mean gray scale level (SD)]	77.2 (17.0)	83.9 (14.8)	0.094	60.2 (46.4–74.0)
TA absolute echo intensity [mean gray scale level (SD)]	88.6 (12.4)	94.6 (12.1)	0.046*	60.8 (47.4–74.1)
*Z*-score echo intensity				
BB *z*-score echo intensity [mean (SD)]	0.5 (1.4)	0.7 (1.9)	0.519	56.3 (42.8–69.9)
FCR *z*-score echo intensity [mean (SD)]	1.4 (1.6)	1.9 (2.0)	0.207	59.2 (45.2–73.2)
RF *z*-score echo intensity [mean (SD)]	1.0 (1.6)	1.6 (1.8)	0.137	58.7 (45.0–72.4)
TA *z*-score echo intensity [mean (SD)]	1.0 (1.2)	1.5 (1.4)	0.118	59.4 (45.9–72.9)
Echo intensity SD				
BB echo intensity SD [mean gray scale level(SD)]	29.0 (4.0)	28.5 (4.4)	0.585	53.3 (39.6–67.0)
FCR echo intensity SD [mean gray scale level(SD)]	21.3 (3.1)	20.8 (3.9)	0.573	56.2 (42.2–70.2)
RF echo intensity SD [mean gray scale level(SD)]	24.2 (3.3)	23.5 (3.5)	0.398	55.9 (42.0–69.8)
TA echo intensity SD [mean gray scale level (SD)]	25.8 (3.6)	26.2 (3.9)	0.698	54.4 (40.5–68.3)
*Z*-score echo intensity SD				
BB *z*-score echo intensity SD [mean (SD)]	0.3 (1.5)	0.1 (1.5)	0.746	53.3 (39.6–67.0)
FCR *z*-score echo intensity SD [mean (SD)]	0.8 (1.4)	0.6 (1.7)	0.716	55.0 (41.0–69.1)
RF *z*-score echo intensity SD [mean (SD)]	0.1 (1.6)	0.04 (1.4)	0.890	54.2 (36.9–65.5)
TA *z*-score echo intensity SD [mean (SD)]	−0.3 (1.1)	−0.2 (1.4)	0.777	55.0 (41.1–68.9)

*BB* biceps brachii, *FCR* flexor carpi radialis, *RF* rectus femoris, *TA* tibialis anterior

* *P* < 0.05

For echo intensity, median CV of three analyses per muscle was 1.9–5.1%. Echo intensity was higher in TA muscle in ICU-AW patients, but *z*-scores of echo intensity were not different (Table 2). Discrimination between patients with ICU-AW and patients without ICU-AW based on echo intensity was poor and failed on *z*-scores of echo intensity.

Sensitivity, specificity, PPV and NPV based on *z*-score cutoff values are presented in Table 3. Specificity was high, but sensitivity, PPV and NPV were all low.

**Table 3:** Sensitivity, specificity, positive predictive value (PPV) and negative predictive value (NPV) for z-score cutoffs of −2 for muscle thickness and +2 for echo intensity

	Cutoff	Sensitivity (%)	Specificity (%)	PPV (%)	NPV (%)
BB thickness	*Z*-score < −2	3	97	50	43
FCR thickness	*Z*-score < −2	27	83	67	48
RF thickness	*Z*-score < −2	3	97	50	43
TA thickness	*Z*-score < −2	8	93	60	43
BB echo intensity	*Z*-score > 2	36	67	58	44
FCR echo intensity	*Z*-score > 2	46	73	68	52
RF echo intensity	*Z*-score > 2	36	67	58	44
TA echo intensity	*Z*-score > 2	38	80	71	49

Echo intensity SD and *z*-scores did not differ between patients with and without ICU-AW, and discrimination failed.

A composite outcome of amount of muscles with a thickness *z*-score of <−2 did not correctly classify patients [ROC–AUC 54.6% (95% CI 43.7–65.5%)], nor did the amount of muscles with an echo intensity *z*-score > 2 [ROC–AUC 56.8% (95% CI 43.4–70.2%)].

### Nerve ultrasound

CSA of the median nerve at the wrist was lower in ICU-AW patients, but showed poor discrimination (Table 4). The other nerve CSA and thickness measures were not different and discrimination failed. Nerve vascularization did not differ between patients with and without ICU-AW.

**Table 4:** Univariate analysis and area under the receiver operating characteristic curve (ROC–AUC) of nerve parameters

	No ICU-AW	ICU-AW	*P* value	ROC–AUC (95% CI)
Median nerve				
CSA wrist [mean mm^2^ (SD)]	10.7 (3.5)	8.9 (2.1)	0.020*	66.7 (53.6–79.9)
CSA proximal [mean mm^2^ (SD)]	7.3 (1.8)	7.6 (1.7)	0.445	52.5 (38.6–66.4)
Thickness proximal [mean mm (SD)]	2.4 (0.4)	2.4 (0.4)	0.977	51.0 (37.2–65.2)
Intraneural vascularization proximal (%)^a^	6 (20.0)	10 (25.6)	0.793	
Peroneal nerve				
CSA fibular head [mean mm^2^ (SD)]	10.8 (3.5)	11.7 (5.0)	0.504	53.1 (35.6–70.7)
CSA knee fold [mean mm^2^(SD)]	8.2 (3.3)	8.2 (3.0)	0.999	52.4 (36.9–67.9)
Thickness knee fold [mean mm (SD)]	2.2 (0.4)	2.3 (0.4)	0.505	54.6 (38.9–70.2)
Intraneural vascularization proximal (%)^a^	3 (12.0)	4 (14.3)	1.000	

* *P* < 0.05

^a^In transversal or longitudinal plane

## Discussion

This study showed that the diagnostic accuracy of quantitative NMUS for diagnosis of ICU-AW is poor when assessed at awakening (median 7–9 days after ICU admission). A single or composite NMUS measurement cannot distinguish between patients with or without ICU-AW. For a *z*-score cutoff of −2 for muscle thickness and +2 for muscle echo intensity (corresponding to values found in 2.3% of healthy individuals), specificity was high, but sensitivity and PPV and NPV were low.

### Muscle thickness and echo intensity

We found that thickness and *z*-scores of thickness of BB and FCR muscles were significantly lower in ICU-AW patients compared to patients without ICU-AW and echo intensity of TA was higher. However, there is a huge overlap of NMUS values of the two groups, causing low diagnostic accuracy. We also found that most *z*-scores were between −2 and +2, which is usually considered the normal range (corresponding to values found in 95% of healthy people).

More time may be needed for muscle thickness to decrease and for echo intensity to increase. Long-term studies show that increased time in the ICU is associated with a substantial reduction in muscle thickness (up to 17.7–30.4% at day 10 after ICU admission [17, 21] and 38.9% after 4 weeks [19]). However, muscle atrophy is also seen in healthy volunteers after bed rest and might not discriminate between patients with and without ICU-AW [31]. Moreover, echo intensity may increase more slowly. The process of recovery of injured muscle tissue, giving an increase in fibrous and/or fat tissue in muscle, may not be detectable in the first weeks after initial muscle injury [32]. However, inflammation may be detectable earlier. In patients with severe sepsis, semiquantitatively graded echo intensity was already significantly higher at day 4 after admission compared to controls [22].

To determine diagnostic accuracy, we chose the moment of awakening, because it allowed a cross-sectional design with direct comparison to strength measurements. However, a diagnosis of ICU-AW before awakening (before muscle strength measurements are possible) is more desirable, because an early diagnosis is a prerequisite for any future preventive measure or treatment to be implemented. Since we found that diagnostic accuracy of NMUS at awakening was poor, it is less likely that differences in thickness and echo intensity will be more noticeable before this time point. Besides, muscle thickness and echo intensity can be influenced by confounding factors, like excessive fluid administration, often present in the first days after ICU admission, impairing the use of NMUS for early diagnosis of ICU-AW.

Alternatively, it might be that not the values at one time point, but the rate at which muscle size decreases or echo intensity increases can discriminate between ICU-AW and no ICU-AW. This would require multiple assessments with ultrasound in the first days after ICU admission to acquire an early diagnosis. Whether the changes in muscle thickness or echo intensity in the first days after admission would be evident enough to discriminate between ICU-AW and no ICU-AW is unknown. Studies with serial NMUS measurements within the first week after admission do not show uniform results: some studies showed a decrease in muscle mass at day 7 after ICU admission, varying from 12.1% [15] to 6.0–24.9% [17], while others did not find changes from baseline in the first week after ICU admission [16, 33]. Hence, the decrease in muscle thickness might be limited in the first week and might become more apparent thereafter. The increase in echo intensity is also more obvious after 7 days on the ICU [16, 17]. Differences between patients with and without ICU-AW were not assessed in these studies.

### Nerve ultrasound in the ICU

To our best knowledge, nerve ultrasound has never been investigated before in ICU patients. Diagnostic accuracy of nerve thickness or CSA for diagnosing ICU-AW in our study was poor. The CSA of the median nerve measured at the carpal tunnel at the wrist was smaller in ICU-AW patients, and other nerve CSA or thickness parameters were not different. This at least indicates that nerve thickness is not increased in ICU-AW when compared to patients without ICU-AW, which is in line with an axonal neuropathy, since nerve thickness is often increased in demyelinating polyneuropathies [8].

It is hypothesized that nerve damage in ICU-AW may be caused by increased vascular permeability causing endoneurial edema and subsequent hypoxia [34]. To compensate, perineural veins may dilate and cause hyperemia and hypervascularization, which could be detected by NMUS [35]. We found hypervascularization in the median nerve in 16 patients and in the peroneal nerve in 7 patients. However, there were no differences between patients with and without ICU-AW.

### Strengths and weaknesses

This is the first study investigating the diagnostic accuracy of quantitative NMUS for diagnosing ICU-AW, and also the first study ever to investigate quantitative nerve ultrasound in ICU patients. We used muscle strength testing as the reference standard, which is a clinically relevant reference standard and recommended method to diagnose ICU-AW [4]. A large number of patients were included, and muscles and nerves were investigated with ultrasound in a systematic and reproducible way. The use of *z*-scores made the comparisons of measurements more reliable because age, gender and muscle side can influence muscle thickness and echo intensity.

As a first step to determine diagnostic accuracy, we investigated NMUS parameters only at the first time point on which the reference standard (MRC score) was available. A limitation is that we did not perform NMUS measurements before or after that time point. We can therefore not rule out that a change in thickness or echo intensity over time may have a good diagnostic accuracy. Additionally, we did not investigate muscle CSA as a measure of muscle mass in our study. Furthermore, NMUS and strength assessment was performed approximately 2 days later in ICU-AW patients compared to patients without ICU-AW. As there was more time in the ICU-AW group for muscle thickness to decrease and echo intensity to increase, this may have potentially overrated the difference with the group without ICU-AW. Given the already low diagnostic accuracy found, this only strengthens our conclusions. Additionally, it might be that muscles that were not assessed in our study may be more sensitive to changes in thickness or echo intensity. Moreover, muscle biopsy or electrophysiological recordings were not performed in our study, because these are not routinely performed in clinical practice to diagnose ICU-AW in our ICU. Therefore, we cannot discriminate between CIP, CIM and CINM in this study.

Although the investigators performing the NMUS were blinded for the exact MRC scores, total blinding to muscle strength is not possible, since the presence or absence of spontaneous movements of the patient already gives an impression of the muscle strength. Additionally, except for intra-rater CV of echo intensity measurements, we did not assess inter- and intra-rater variability.

A limitation of quantitative NMUS in general is the fact that it is complicated to directly compare NMUS results between studies, because of differences in image acquisition settings, probe position, ultrasound machines, etc. Gray scale levels are specific for the ultrasound machine used and cannot be compared to data obtained by other ultrasound machines unless calibrated [8]. Calibration can be done with a universal phantom.

## Conclusion

A single neuromuscular ultrasound at the moment a patient awakens does not discriminate between patients with and without ICU-AW.

## Additional file

**Additional file 1: Figure E1.** Muscle measurements predefined measurement sites. **Table E1.** Muscle measurements predefined measurement sites. **Figure E2.** Ultrasound image of the rectus femoris. **Table E2.** Regression model formulas for normal values of muscle thickness and muscle echo intensity. **Table E3.** Characteristics of new healthy control cohort. **Figure E3.** Muscle thickness and echo intensity.
