# Supplementary material for: Diagnostic accuracy of quantitative neuromuscular ultrasound for the diagnosis of intensive care unit-acquired weakness: a cross-sectional observational study
Source: Ann Intensive Care. 2017 Apr 5;7:40. doi: 10.1186/s13613-017-0263-8 (PMC5382120; doi:10.1186/s13613-017-0263-8)
Supplement: Supplementary file 1 — Additional file 1: Figure E1. Muscle measurements predefined measurement sites. Table E1. Muscle measurements predefined measurement sites. Figure E2. Ultrasound image of the rectus femoris. Table E2. Regression model formulas for normal values of muscle thickness and muscle echo intensity. Table E3. Characteristics of new healthy control cohort. Figure E3. Muscle thickness and echo intensity. [file 13613_2017_263_MOESM1_ESM.doc]

**Supplemental Data file**

**Diagnostic accuracy of quantitative neuromuscular ultrasound for the diagnosis of intensive care unit-acquired weakness: a cross-sectional observational study.**

Esther Witteveen, Juultje Sommers, Luuk Wieske, Jonne Doorduin, Nens van Alfen, Marcus J. Schultz, Ivo N. van Schaik, Janneke Horn, Camiel Verhamme

- **Figure E1. Muscle measurements predefined measurement sites**
- **Table E1. Muscle measurements predefined measurement sites**
- **Figure E2. Ultrasound image of the rectus femoris**
- **Table E2. Regression model formulas for normal values of muscle thickness and muscle echo intensity**
- **Table E3. Characteristics new healthy control cohort**
- **Figure E3. Muscle thickness and echo intensity**

**Figure E1. Muscle measurements predefined measurement sites**

If ultrasound was not possible on the preferred side, for example due to arterial lines or dressings, the opposite side was studied.


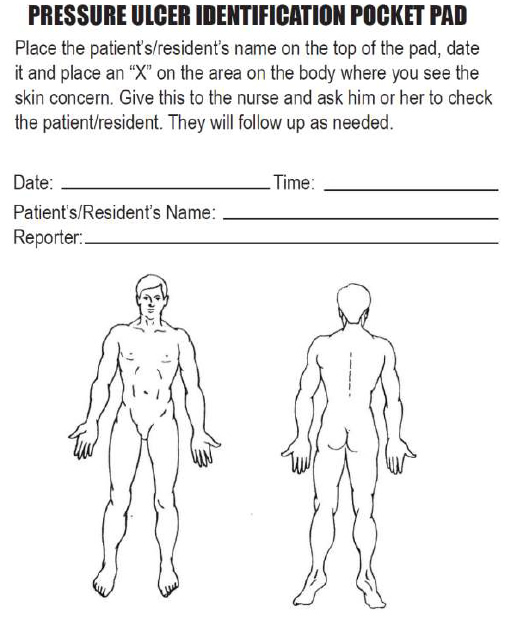


**Table E1. Muscle measurements predefined measurement sites**

|  | **Biceps Brachii**  **(BB)** | **Flexor carpi radialis**  **(FCR)** | **Rectus femoris**  **(RF)** | **Tibialis anterior (TA)** |  |
| --- | --- | --- | --- | --- | --- |
| Preferred side | Left | Right | Right | Left |  |
| Measurement site | 2/3 distance from acromion to antecubital crease | 1/3 distance from antecubital fold to distal radius (muscle has triangular shape) | Halfway from anterior superior iliac spine and superior border of patella | 1/3 distance from inferior border of patella to the lateral malleolus |  |
| Correct transducer position | Humerus located in the middle and neurovascular bundle on the edge of the picture | Horizontal and flat position of radius and ulna.  15˚ angle between radius and ulna | Femur located in the middle with vastus intermedius and rectus femoris visible above the femur | Tibial cortex at about 45˚ angle.  Central fascia is evident |  |
| Muscle thickness measurement | From humerus to upper border of BB (including brachialis muscle) | From deepest point of FCR to upper border of FCR | From femoral bone to upper border of RF (including vastus intermedius muscle) | From lower border of TA to upper border |  |
| Caliper positions for thickness and subcutaneous tissue | 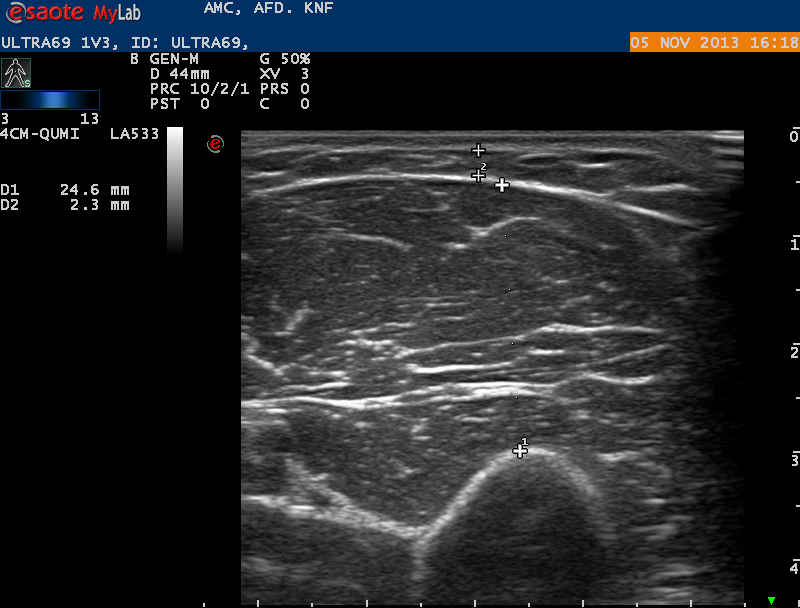 | 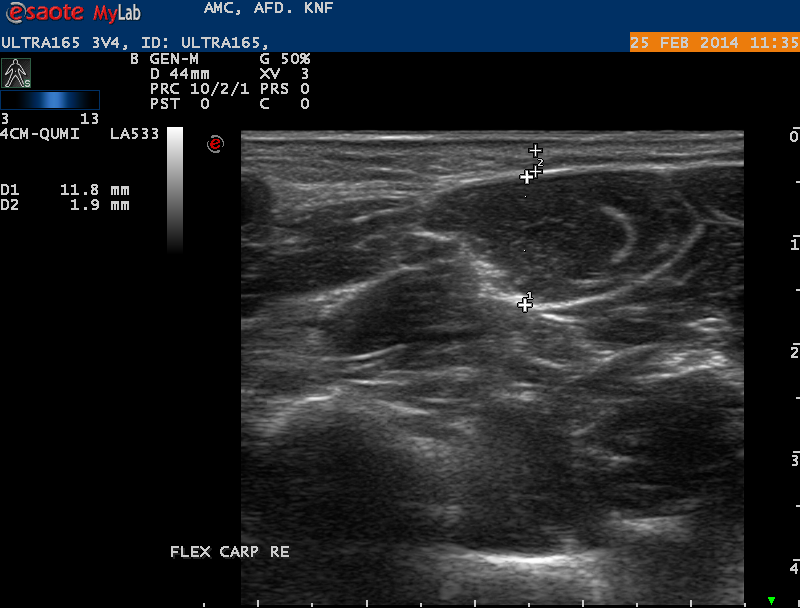 | 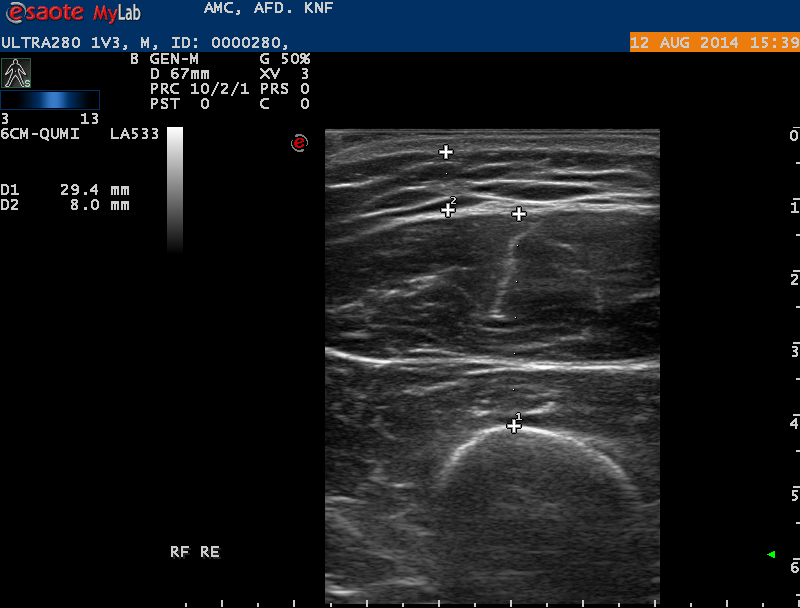 | 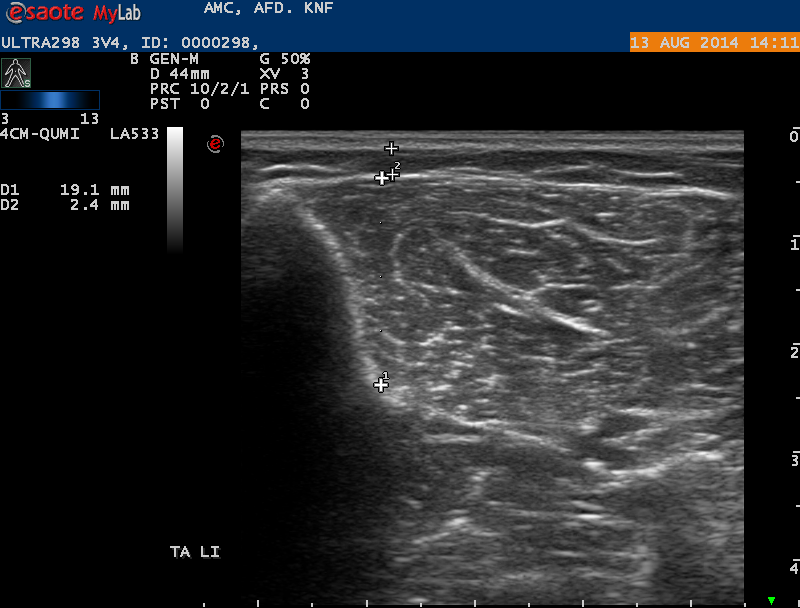 |  |
| Region of interest; lateral borders removed automatically | 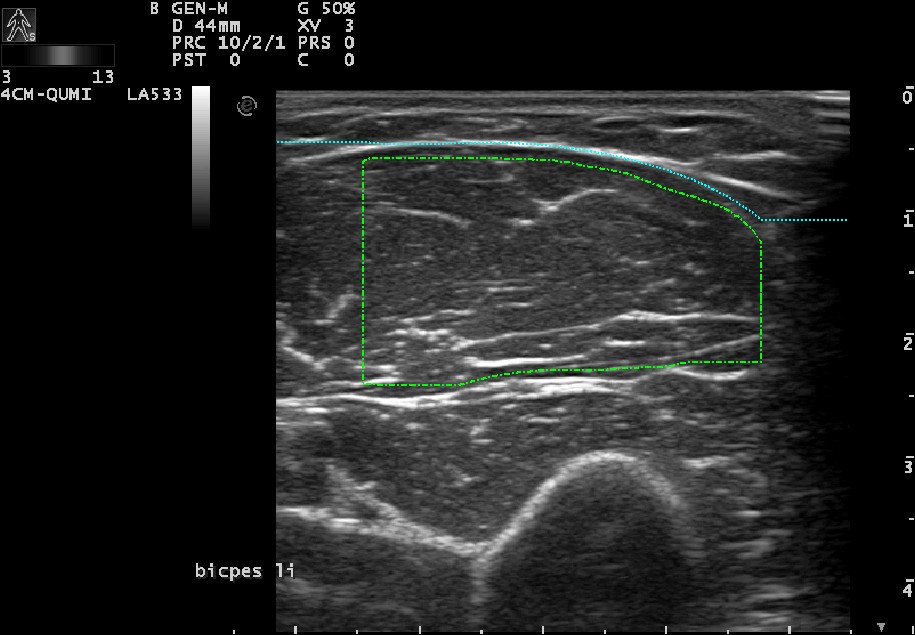 | 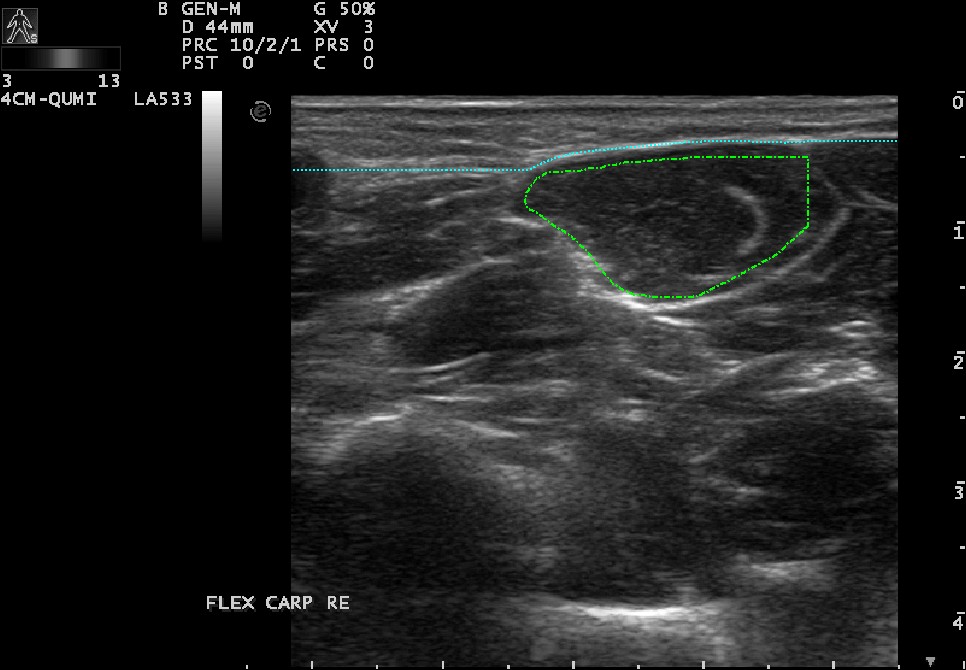 | 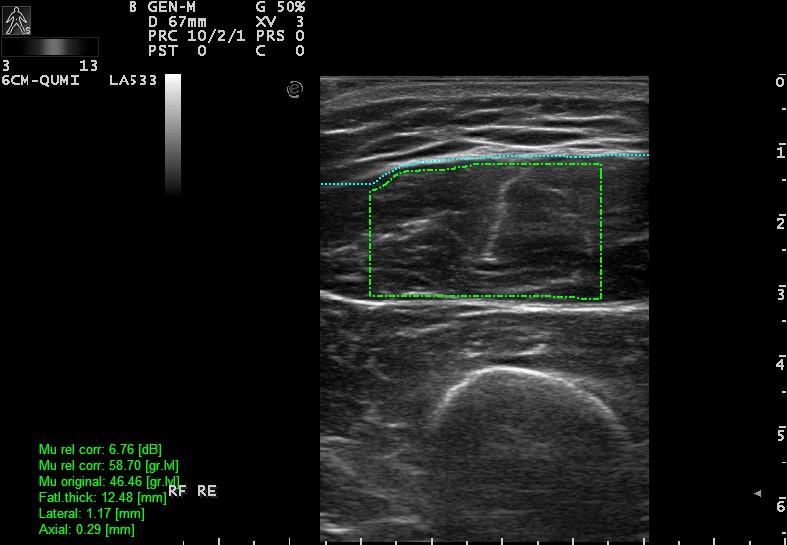 | 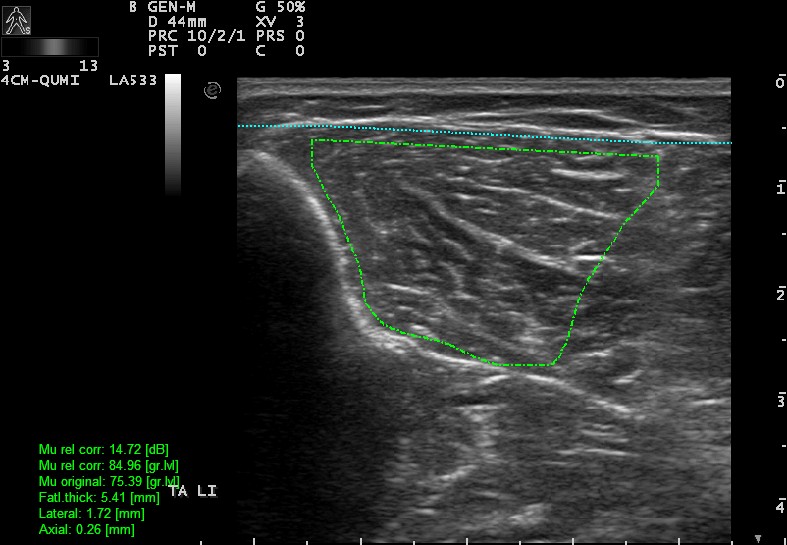 |  |

**Figure E2. Ultrasound image of the rectus femoris**

Ultrasound image of the rectus femoris (RF) muscle with calipers placed for muscle thickness (including the vastus intermedius muscle (VI)) and subcutaneous tissue (left picture), and region of interest (ROI) drawn (right picture). F=femur.


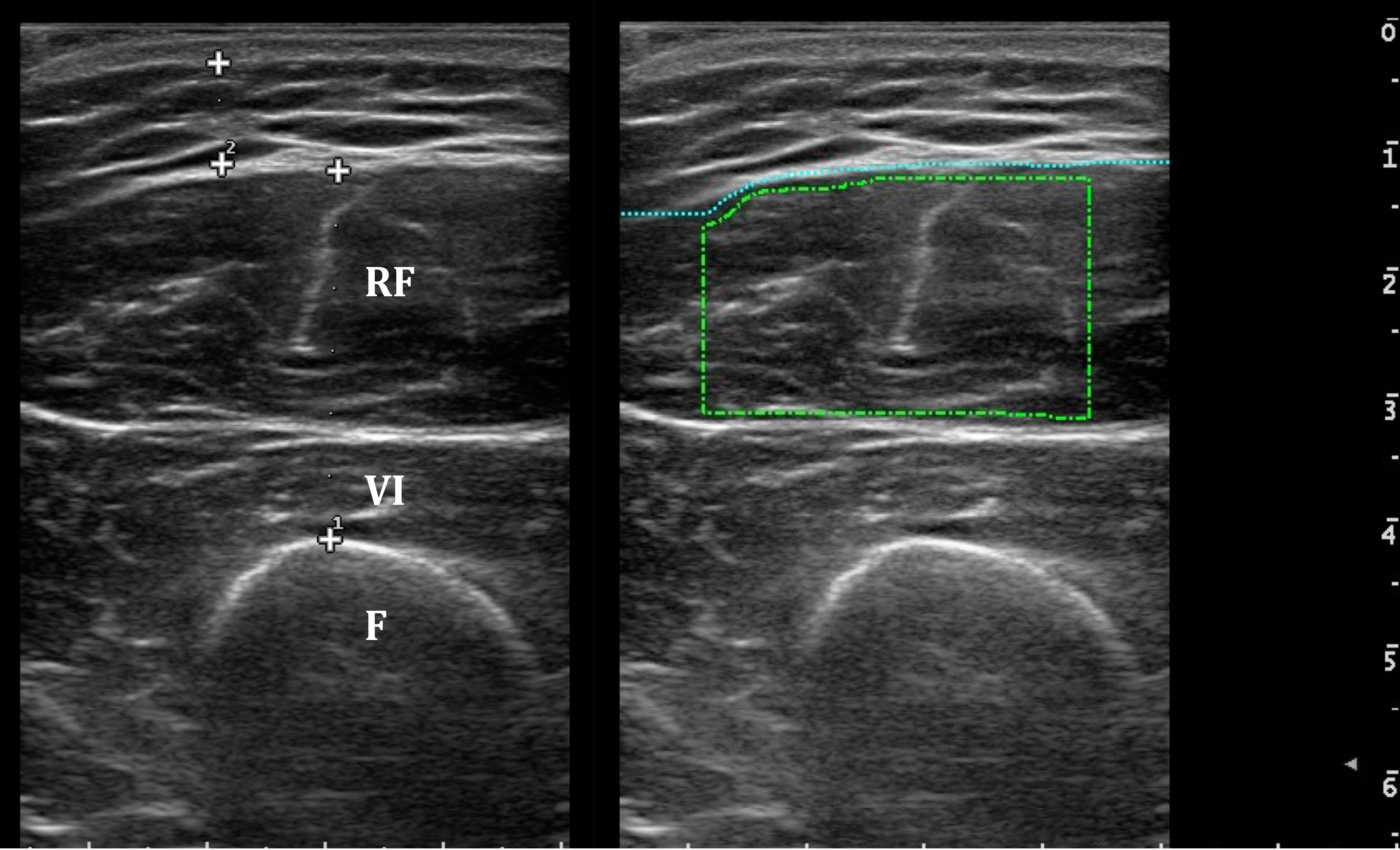


**Table E2. Regression model formulas for normal values of muscle thickness and muscle echo intensity**

| **Muscle** | **Sex** | **Age** | **Side** | **Regression model**  **thickness** | **Regression model**  **echo intensity** |
| --- | --- | --- | --- | --- | --- |
| BB | M | 18-99 | D | 0.415+0.047*A-8.07*10^-4^*A²+3.79*10^-6^*A³-0.071*S | 64.337+3.617*10^-5^*A^3^ |
|  |  |  | Non-D |  | 48.311+0.4*A |
|  | F | 18-99 | D | 0.754+1.68*10^-4^* A²-1.68*10^-4^*A³-0.081*S | 61.432+3.391*10^-3^*A^2^ |
|  |  |  | Non-D |  | 62.594+3.853*10^-5^*A^3^ |
| FCR | M | 18-99 | D | 0.762+0.007*W | 46.951+2.192 *10^-5^*A^3^ |
|  |  |  | Non-D | 0.825+0.007*W | 40.965+0.179*A |
|  | F | 18-81 | D | 1.11 | 57.197 |
|  |  | 81-99 |  |  | 46.951+2.129*10^-5^*A^3^ |
|  | F | 18-81 | Non-D | 0.388+0.03*BMI | 56.727 |
|  |  | 81-99 |  |  | 10.965+ 0.179*A |
| RF | M | 18-25 | Both | 1.518-8.31*10^-7^*A³ | 55.827 |
|  |  | 25-99 |  |  | 47.917 +0.332*A |
|  | F | 18-99 | Both | 3.985-0.005*A-0.015*L | 81.507+0.283*A-0.357*W |
| TA | M | 18-99 | Both | 0.959-1.9*10^-7^*A³ | 59.65+0.301*A |
|  | F | 18-99 | Both | 2.16 | 67.17+0.29*A |

BB=biceps brachii, FCR=flexor carpi radialis, RF=rectus femoris, TA=tibialis anterior, M=male, F=female, D=dominant, Non-D= non-dominant, A=age in years, S=side (right=1, left=2), W=weight in kg, BMI=body mass index, L=length in cm

Thickness regression models for BB and TA muscles were acquired from Arts et al. (Muscle and Nerve 2010) and the thickness regression models of RF muscles from Nijboer et al. (Muscle and Nerve 2011).

Thickness regression models of FCR and all echo intensity regression models were acquired from a new healthy control cohort from the Radboud University Medical Center Nijmegen (see table E3).

**Table E3 Characteristics new healthy control cohort**

Characteristics of new healthy control cohort of Radboud University Medical Center Nijmegen

| **Age group** | **Number** | | **Height (cm)** | | **Weight (kg)** | | **Body Mass index (BMI)** | |
| --- | --- | --- | --- | --- | --- | --- | --- | --- |
|  | M | F | M | F | M | F | M | F |
| 18-30 | 6 | 7 | 184.0 (182.3-187.3) | 167.0 (166.0-169.0) | 74.0 (72.3-78.8) | 60.0 (54.5-62.0) | 21.9 (21.0-23.1) | 22.0 (19.6-22.7) |
| 30-40 | 4 | 5 | 186.5 (183.3-187.8) | 173.0 (168.0-176.0) | 75.0 (72.0-82.8) | 70.0 (60.0-72.0) | 22.6 (21.4-24.6) | 22.9 (21.6-24.1) |
| 40-50 | 6 | 3 | 180.0 (180.0-183.8) | 174.0 (170.5-182.0) | 77.0 (74.8-77.0) | 69.0 (67.0-93.5) | 23.8 (23.1-24.3) | 24.7 (23.1-28.7) |
| 50-60 | 6 | 7 | 176.5 (172.8-178.8) | 171.0 (165.5-172.0) | 69.5 (68.3-71.5) | 66.0 (61.0-68.0) | 23.0 (21.9-24.0) | 22.5 (22.0- 23.2) |
| 60-70 | 4 | 7 | 179.0 (174.5-182.3) | 171.0 (160.0-172.8) | 95.5 (84.0-99.8) | 76.0 (69.5-82.0) | 29.2 (26.1-30.9) | 25.1 (24.6-28.3) |
| 70-80 | 4 | 5 | 183.0 (175.3-184.5) | 170.0 (168.0-172.0) | 83.5 (76.0-85.8) | 71.0 (69.0-74.0) | 24.4 (24.2-25.1) | 24.4 (23.7-25.6) |
| >80 | 2 | 2 | 166.5 (166.3-166.8) | 176.0 (174.5-177.5) | 70.5 (68.7-72.3) | 77.5 (76.3-78.8) | 25.4 (24.9-26.0) | 25.1 (24.2-25.9) |

M=male, F=Female

**Figure E3. Muscle thickness and echo intensity**

Z-scores of muscle thickness (A) and echo intensity (B) for four muscles.

Crossbars represent mean values. The shaded area is the area from z-score -2 to

+2, the area in which 95% of the values of healthy people are. Differences are

analyzed using Welch’s t test and p-values are expressed.


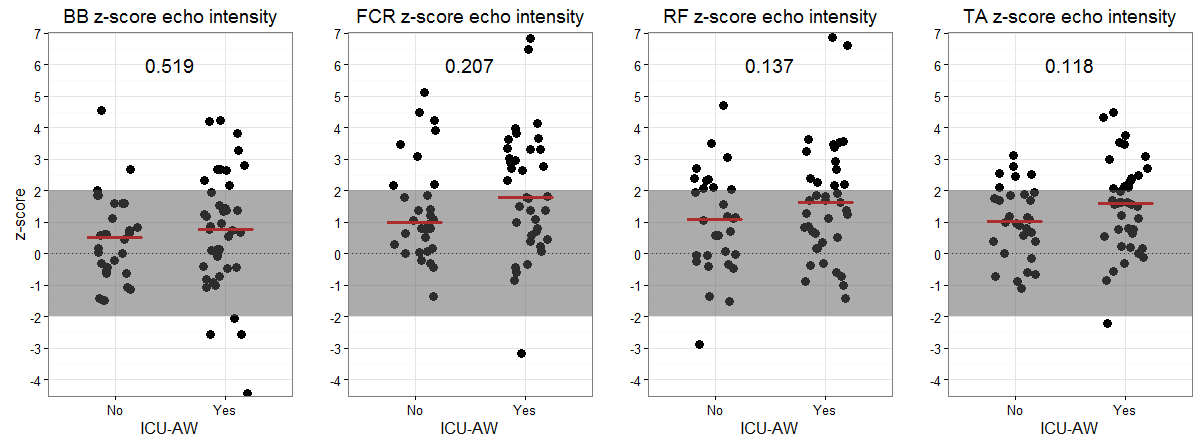

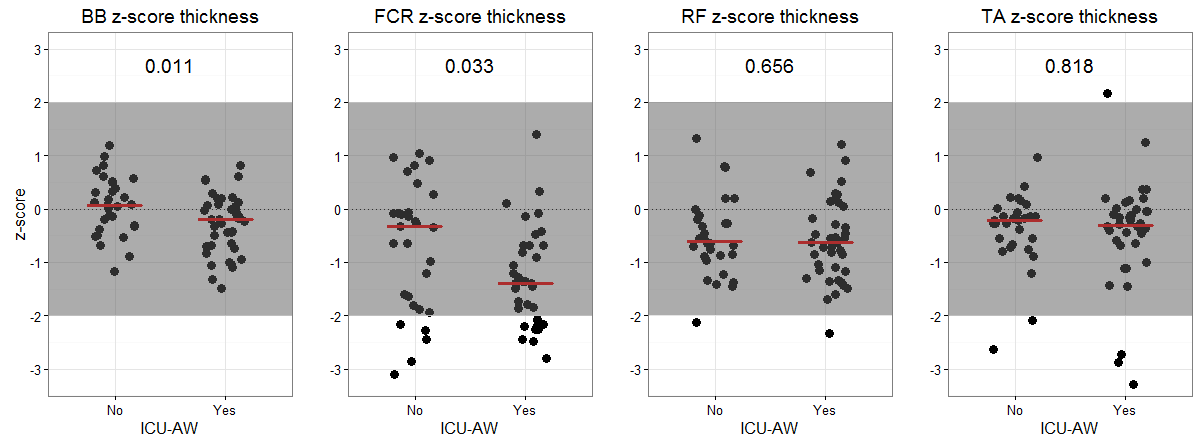


**A**

**B**

BB=biceps brachii, FCR=flexor carpi radialis, RF=rectus femoris, TA=tibialis anterior, ICU-AW=ICU-acquired weakness
